# Imaging methods in the assessment of nonalcoholic fatty liver disease

**DOI:** 10.1590/0100-3984.2020.53.2e3

**Published:** 2020

**Authors:** Daniella Braz Parente

**Affiliations:** 1 Universidade Federal do Rio de Janeiro (UFRJ), Rio de Janeiro, RJ, Brazil. Email: daniella.parente@gmail.com.

Nonalcoholic fatty liver disease (NAFLD) is a public health problem that affects one third of the world population. In parallel with the obesity epidemic, the incidence of NAFLD has been increasing worldwide, in all age groups, including children, among all ethnicities, and in all socioeconomic groups^(1,2)^. In the United States, NAFLD is already the second leading indication for liver transplantation^(2)^.

The presentation of NAFLD is a spectrum that begins with isolated steatosis, which is associated with increased cardiovascular risk and an increased incidence of colorectal cancer^(3)^. Approximately 25% of patients with isolated steatosis progress to nonalcoholic steatohepatitis (NASH), a condition that presents a higher risk to progress to chronic liver disease. The higher the degree of steatosis, greater the risk to develop NASH. Approximately 25% of patients with NASH progress to chronic liver disease, with fibrosis, cirrhosis, and risk of complications, that includes portal hypertension and hepatocellular carcinoma^(2,4)^.

Although liver biopsy is still the only method capable to diagnose NASH^(5)^, it is not suitable for follow-up of NAFLD, a condition that affects one third of the population^(6,7)^, for many reasons: its invasive nature; the associated risk of morbidity and mortality; the heterogeneity in the distribution of fibrosis in the liver parenchyma; the interobserver and intraobserver variability; the cost; and poor patient and physician acceptance. Therefore, noninvasive methods to diagnose NASH are necessary, including serological biomarkers, in combination with clinical characteristics, anthropometric measurements, other biochemical parameters, and imaging findings^(8-10)^.

Various imaging methods have been used to diagnose and monitor NAFLD, such as ultrasound, computed tomography (CT), magnetic resonance imaging (MRI) and, recently, the controlled attenuation parameter (CAP), which is determined in conjunction with transient elastography, and magnetic resonance elastography (MRE). Although ultrasound has the advantage of being widely available, it does not have high interobserver reproducibility and is not sensitive enough to detect mild steatosis. Mild steatosis is also undetectable on CT, which, together with the exposure of patients to radiation, makes CT a poor method for follow-up. Although access to MRI is more limited, the method has high reproducibility with multi-echo fat quantification techniques and proton spectroscopy^(11-13)^.

CAP is a new method that evaluates hepatic steatosis in a quantitative way and has been widely used by hepatologists, because it is easily used at the bedside and has the advantage to be associated with transient elastography, which measures liver stiffness. Thus, the use of CAP combines information on steatosis and fibrosis in a single method^(11)^. However, CAP cutoff values are still widely disputed, not only for the diagnosis of steatosis but also for its degrees into mild, moderate, and severe forms^(14,15)^. In patients with NAFLD, the cut-off points for fibrosis, its degrees, and the relationship with the METAVIR fibrosis score are also not well established^(15,16)^. Obese patients and patients with ascites are also difficult to assess by transient elastography. The use of the FibroScan XL probe has improved the performance of CAP in obese patients^(17)^.

The advantage of MRE is that it assesses a larger area of the liver parenchyma. Given that fibrosis has a heterogeneous distribution, liver stiffness may undergo variations that would be better evaluated by MRE. Like CAP determination during transient elastography, MRE allows the measurement of liver stiffness to be associated with the liver fat fraction. In addition, the use of intravenous contrast allows the entire liver to be assessed, making it possible to diagnose focal lesions^(11)^.

A number of factors influence the measurement of liver stiffness on ultrasound elastography and MRE. It is essential that proper examination technique be employed: measurements should be taken during neutral breath hold, because deep inhalation and exhalation significantly alter the results. Other factors, such as inadequate fasting, elevated aminotransferases, liver congestion, liver inflammation, and alcohol consumption, also increase liver stiffness. Therefore, elevated values should be correlated with clinical and biochemical parameters^(11)^.

In the previous issue of **Radiologia Brasileira**, Silva et al.^(18)^ presented an excellent review on ultrasound elastography in patients with hepatic steatosis, comprehensively addressing the various methods, as well as their advantages and disadvantages, concluding, in an elegant manner, with what is established in the literature. Their article calls attention to NAFLD as a major public health problem and discusses the spectrum of the disease, as well as the complexity, difficulties, and methods for diagnosis.
